# Using multi-level Bayesian lesion-symptom mapping to probe the body-part-specificity of gesture imitation skills

**DOI:** 10.1016/j.neuroimage.2017.08.036

**Published:** 2017-11-01

**Authors:** Elisabeth I.S. Achilles, Peter H. Weiss, Gereon R. Fink, Ellen Binder, Cathy J. Price, Thomas M.H. Hope

**Affiliations:** a Department of Neurology, University Hospital of Cologne, Cologne, Germany; b Cognitive Neuroscience, Institute of Neuroscience and Medicine (INM-3), Research Centre Jülich, Germany; c Wellcome Trust Centre for Neuroimaging, University College London, UK

**Keywords:** Stroke, Lesion, Imitation, Apraxia, Bayesian

## Abstract

Past attempts to identify the neural substrates of hand and finger imitation skills in the left hemisphere of the brain have yielded inconsistent results. Here, we analyse those associations in a large sample of 257 left hemisphere stroke patients. By introducing novel Bayesian methods, we characterise lesion symptom associations at three levels: the voxel-level, the single-region level (using anatomically defined regions), and the region-pair level. The results are inconsistent across those three levels and we argue that each level of analysis makes assumptions which constrain the results it can produce. Regardless of the inconsistencies across levels, and contrary to past studies which implicated differential neural substrates for hand and finger imitation, we find no consistent voxels or regions, where damage affects one imitation skill and not the other, at any of the three analysis levels. Our novel Bayesian approach indicates that any apparent differences appear to be driven by an increased sensitivity of hand imitation skills to lesions that also impair finger imitation. In our analyses, the results of the highest level of analysis (region-pairs) emphasise a role of the primary somatosensory and motor cortices, and the occipital lobe in imitation. We argue that this emphasis supports an account of both imitation tasks based on direct sensor-motor connections, which throws doubt on past accounts which imply the need for an intermediate (e.g. body-part-coding) system of representation.

## Highlights

• Novel Bayesian lesion-symptom analyses are proposed.
• Different levels of analysis emphasise different lesion sites for gesture imitation deficits.
• All levels argue against the body-part-specificity of gesture imitation skills.
• A direct sensor-to-motor pathway model of gesture imitation is supported.

## 1 Introduction

Even within the first few weeks after birth, infants appear to be able to imitate certain facial and manual gestures (Meltzoff and Moore, 1977). These apparently hard-wired skills (Meltzoff and Moore, 1977, Meltzoff and Moore, 1997) may provide the foundation for much of our subsequent learning, including language acquisition, socialisation and enculturation (Brass and Heyes, 2005). Clues to the neural substrates of imitation skills can be garnered by localizing the brain damage which disrupts them. Deficits of imitation skills are a common symptom of apraxia, a disorder of motor cognition which most often occurs after left hemisphere (LH) stroke (Donkervoort et al., 2000), and which cannot be explained by primary deficits of the sensor-motor system or disturbed communication (Dovern et al., 2012). Past studies of apraxic patients suggest that there is a body-part-specific distribution of imitation skills across the two hemispheres of the brain. Hemispheric asymmetries in damage-deficit associations have been reported for postures of the upper versus lower face or of the fingers and feet versus hand (Bizzozero et al., 2000, Goldenberg and Strauss, 2002). LH damage can impair all of these skills, whereas right hemisphere (RH) damage appears only to impair a subset (upper face, feet and fingers: (Goldenberg and Karnath, 2006)).

While these hemispheric asymmetries in imitation skills are well confirmed, analogous distinctions within the LH are still debated. Some of the earliest evidence in favour of body-part-specific mechanisms within the left hemisphere causing a dissociation between hand and finger imitation skills was reported by Haaland and colleagues (Haaland et al., 2000), who tested 41 stroke patients' abilities to imitate gestures combining finger and hand postures, but distinguished between “target errors” of hand position and “internal hand position” errors of finger postures. Hand position errors were found in most (4/5) patients whose lesions were exclusively posterior to the central sulcus, and in none whose lesions were exclusively anterior to the same sulcus (n = 4), whereas finger position errors were found in all of those same patients with exclusively anterior lesions (n = 4), and in 3/5 with posterior lesions. Though somewhat equivocal, this anterior (finger)/posterior (hand) dissociation is consistent with the results of a later lesion subtraction analysis (with 44 patients), which associated disturbed imitation of finger postures with lesions anterior to the central sulcus including the opercular portion of the inferior frontal gyrus (IFG), and disturbed imitation of hand postures with lesions posterior to the central sulcus affecting the left inferior parietal lobe (IPL) and the temporo-parieto-occipital junction (Goldenberg and Karnath, 2006). This latter study also goes further than that by Haaland and colleagues, reporting both a behavioural and a neuroanatomical double dissociation between hand and finger imitation skills.

Evidence for an apparently similar posterior (hand)/anterior (finger) dissociation was also reported in a more recent study employing voxel-based lesion symptom mapping (VLSM) in 43 LH stroke patients, associating deficits of hand imitation with lesions of the inferior and superior parietal cortex, and deficits of finger imitation with smaller frontal regions (Dovern et al., 2011). However, these authors also associated finger imitation deficits with inferior parietal lesions, posterior to the central sulcus. Moreover, there was no evidence at all for a posterior/anterior distinction in a recent VLSM study with a larger sample of 96 acute LH stroke patients by Hoeren and colleagues, which associated both types of deficit with lesions of the posterior inferior parietal lobe (Hoeren et al., 2014). Unlike the other studies mentioned so far, this latter work also went beyond a descriptive comparison of the lesions associated with one or the other deficit (Gelman and Stern, 2006), and probed for deficit-by-lesion-location interactions (henceforth ‘interactions’) more formally. Their results suggest that damage to the left lateral occipito-temporal cortex was associated with relatively greater impairments of hand than finger imitation, but no reverse interaction was found (i.e. no locations where damage was associated with greater deficits in finger than hand imitation). However, these authors found no significant voxels at all when lesion volume was controlled, which raises the concern that there is a confound at play here, with the apparent interaction potentially driven by lesion volume differences, perhaps only accidentally correlated with damage to the lateral occipito-temporal cortex (Karnath and Smith, 2014).

It seems fair to say that these prior studies tell a complex and inconsistent story about the body-part-specificity of gesture imitation. Most studies report only a partial dissociation between hand and finger imitation skills: i.e. damage which impairs finger imitation but not hand imitation (Haaland et al., 2000), or vice versa (Hoeren et al., 2014). The only study, at least that we could find, which reports a full double dissociation between these tasks (Goldenberg and Karnath, 2006), emphasises qualitative methods and has not been replicated in larger samples. One interpretation of these results is that the studies to date have been underpowered. In what follows, we search for task by lesion interactions in a much larger sample of LH stroke patients (n = 257), both to test this notion, and to characterise the effect on the results after controlling for lesion volume (along with two other nuisance covariates: age at onset and time post-stroke). Another interpretation of the result is that there really are no significant associations (or interactions) to be found – either because the neural substrates of the two skills are actually similar, or because voxel-based methods are simply inappropriate to find them. To test this interpretation, we (a) introduce a lesion analysis method based on Bayesian statistics to quantify the evidence both for and against voxel-wise lesion-symptom associations (and interactions), drawing on the logic proposed in (Wetzels and Wagenmakers, 2012); and (b) explore how the evidence for those associations and interactions changes as we ascend hierarchical levels of analysis, from voxels, through anatomically defined brain regions, to pairs of those regions.

## 2 Material and methods

### 2.1 Patient sample

We retrospectively analysed hand and finger imitation scores and lesions of 257 patients who had suffered a single (first ever) unilateral left-hemispheric ischaemic stroke: 82 women; age = 56 ± 14 years; time since stroke at assessment = 33 ± 82 weeks; 75% (194) of the patients were assessed < 6 months post-stroke, and 58% (148) were assessed within a month post-stroke. The data were drawn from a database providing lesion and behavioural information of stroke patients enrolled in previous studies of motor cognition of the University Hospital of Cologne and the Research Centre Jülich. Recruitment sites included the University Hospital of Cologne and the surrounding neurological rehabilitation centres. Other aetiologies than ischaemic strokes such as haemorrhage or tumors were excluded. All patients were right-handed prior to stroke. Furthermore, patients suffering from any other neurological or psychiatric diseases (e.g. depression) were excluded. Subjects were also included if they were between 18 and 80 years old when assessed.

We had only sparse quantitative data concerning the patients' language skills, but they were excluded if they presented with aphasia thought to be severe enough to compromise either their consent to participate, or their understanding of the imitation tasks. Our exclusion only of those patients whose aphasia was so severe that it compromised their grasp of the tasks is consistent with the approach used in (Hoeren et al., 2014), and all of the patients in (Goldenberg and Karnath, 2006) were aphasic. Patients had given written informed consent for participating in the original studies on motor cognition from which these data are drawn from (each of these studies was performed in accordance with the Declaration of Helsinki and was approved by the local ethics committee). Retrospective analyses using these data were approved by the institutional review board.

### 2.2 Testing procedures

All patients were assessed with the test of imitating finger and hand gestures by Goldenberg (1996). Here, the examiner sits opposite to the patient and demonstrates ten hand and ten finger gestures in a mirror like fashion. The examiner uses the hand opposite to the patient's non-paretic ipsilesional hand, which the patient is supposed to use for imitation. After the first demonstration of each gesture, the examiner forms a fist (neutral gesture) and the patient is asked to imitate the previously shown gesture. Two points are allocated for correct imitation, based solely on the final position of the gesture (self-corrections or hesitations do not influence the score). If imitation is incorrect, the examiner repeats the demonstration of the gesture and then returns to the neutral gesture (fist). The patient is asked to imitate the gesture once more. One point is allocated for correct imitation in this second trial, and no points are awarded if the patient fails at the second attempt. A patient is considered to suffer from a hand imitation deficit if the total imitation score for the ten hand gestures is 17 or less of the 20 possible points (two available points for each of the ten gestures) (Goldenberg, 1996). A patient is considered to suffer from a finger imitation deficit if the total imitation score for the ten finger gestures is 16 or less of the 20 possible points (Goldenberg, 1996, Hoeren et al., 2014).

The gestures employed in Goldenberg's test were originally meant to be ‘meaningless’, in the sense that they conveyed no direct semantic content. However, this characterization has been challenged with a recent analysis suggesting that most of the finger gestures can be interpreted as meaningful (Achilles et al., 2016). The difference is important because meaningful and meaningless gestures might be processed differently in the brain (Rumiati et al., 2009), which raises the possibility that any apparently body-part-specific differences that we find might in fact be driven by semantics. We did not attempt to exclude this possibility in the analyses that follow, simply to maximize their comparability with analogous past work. But we note that in both the analyses that follow, and the prior work that inspired them, this ‘semantic confound’ might drive false positive results (i.e. regions where damage appears to impair imitation skills in an effector-specific manner) simply because our measurement tool is confounded by semantics.

### 2.3 Imaging procedures and lesion mapping

Lesion mapping was performed using either clinical MRI (n = 129) or CT (n = 128) scans. The mean time between the date of stroke and the neuropsychological assessment was 33 ± 82 weeks; what we call ‘time post-stroke’ in what follows. The mean time between imaging and behavioural assessment was 17 ± 49 weeks, though this difference was less than a month in 68% (173) of the patients, and including it as a nuisance regressor had no substantive effect on our results.

Lesions were marked manually on axial slices of a T1-weighted template MRI scan from the Montreal Neurological Institute (MNI) using the MRIcron software package with a 1 × 1-mm in-plane resolution. Lesions were mapped onto the slices in steps of 5 mm in MNI space by using the identical or the closest matching axial slices of each individual's CT or MRI, respectively; we found no consistent effects driven by whether patients' lesions were identified from CT or MRI. The lesion slices were convolved into lesion volumes, in MNI space, with a pyramidal kernel, and down-sampled to representation at 2 mm^3^ resolution. Detailed scanning sequences varied across the sample, which is aggregated from several smaller studies.

### 2.4 Lesion-symptom analyses

#### 2.4.1 Voxel-based lesion symptom analyses (VLSM)

We conducted analyses of lesion-symptom associations at the voxel-level for hand and finger imitation deficits separately, and for the difference between them (i.e. hand imitation task score minus finger imitation task score), the latter to test for task specific lesion-symptom interactions. To not only quantify the evidence for but also against the presence of associations, we used a novel, Bayesian VLSM, and compared and contrasted its results to those of a ‘Frequentist’ form of VLSM (Bates et al., 2003).

In the Frequentist analyses, we performed t-tests for independent samples (not assuming equal variance) at each voxel, with groups defined by the presence or absence of damage in each voxel. Correction for multiple comparisons was accomplished both by False Discovery Rate (FDR) correction (Benjamini and Hochberg, 1995), and by permutation thresholding with 1000 permutations, i.e., Family-Wise Error (FWE) correction. We repeated these analyses twice: once with raw scores (/hand minus finger score differences) as the target variables, and once with residuals after regressing out: (a) lesion volume; (b) time post-stroke; and (c) age at stroke onset. Our principal interest here was in the effect of the control for lesion volume, but there were no substantive differences between the results we report controlling for all three covariates and those controlling for lesion volume alone.

In the Bayesian analyses, we implemented a one-way analysis of variance, drawing on the test proposed in (Wetzels et al., 2012), with task score (or the difference between hand minus finger imitation scores) as the dependent variable and groups again defined by the presence or absence of lesion damage in each voxel. Using a slightly more conservative threshold than proposed by Jeffreys' (1961), we define ‘strong’ evidence for or against a lesion-symptom association as evidence at least 20 times stronger than the reverse: i.e. Bayes Factor (BF) > 20 or < $\frac{1}{20}$; log BF > ∼3 or < ∼−3. We use log Bayes Factors here principally for ease of visual illustration in the figures, since the log transform makes the scale symmetrical around zero (log BF = 0 where BF = 1: i.e. where the evidence in favour of the null and alternative hypotheses is perfectly balanced). Both analyses were restricted to the voxels where at least 10% (26) of the patients had damage, and we used a rank-based method to transform all variables to a normal distribution with unit variance; this latter transform ensures that the assumptions implicit in our subsequent analyses will be met. All analyses were implemented in Matlab script, and run on a 6-core desktop PC, running Windows 7.

#### 2.4.2 Region-based lesion-symptom mapping (RLSM)

Recognizing the limits imposed by mass univariate analyses pitched solely at the level of individual voxels (e.g. (Inoue et al., 2014, Mah et al., 2014)), we also ran analyses analogous to VLSM, but pitched at the level of anatomically defined regions, considered both singly and in pairs. These analyses employed region masks drawn from a variety of publically available atlases, including the Anatomy Toolbox (Eickhoff et al., 2005), the Automatic Anatomical Labelling toolbox (Tzourio-Mazoyer et al., 2002), the ICBM-DTI-81 white-matter labels atlas (Oishi et al., 2011) and the JHU white-matter tractography atlas (Hua et al., 2008). Many of these region masks overlapped, but the aim here was not simply to parcellate the brain; rather, we were attempting to group the tested voxels in a flexible manner, without prejudging which anatomical regions, and what level of resolution, might be most relevant to either imitation skill. Where a given atlas supplied region masks in a probabilistic format, we thresholded the mask to include only those voxels where the region was present in at least 25% of participants.

Armed with our set of region masks, we calculated the proportion of each mask that was destroyed by each patient's lesion (the ‘lesion load’): 0% if the mask was completely preserved, rising to 100% when the mask was completely destroyed by (i.e. contained within) a given lesion. We considered only those regions where at least 10% (26) of the patients' lesions destroyed at least some part of that region. The 122 region masks that met this threshold excluded the cerebellum, but otherwise covered most of the left hemisphere of the brain (see Fig. S1). We then used correlation to quantify the associations between lesion load and task scores in each of these 122 regions. We repeated this analysis for both hand and finger imitation scores separately, and also for the within-subject differences between them (as in the VLSM analyses). And as in the VLSM analyses, we quantified the evidence for and against correlations using a Bayesian test, as described in (Wetzels and Wagenmakers, 2012).

## 3 Results

### 3.1 Lesion and behavioural data

**Fig. 1:**
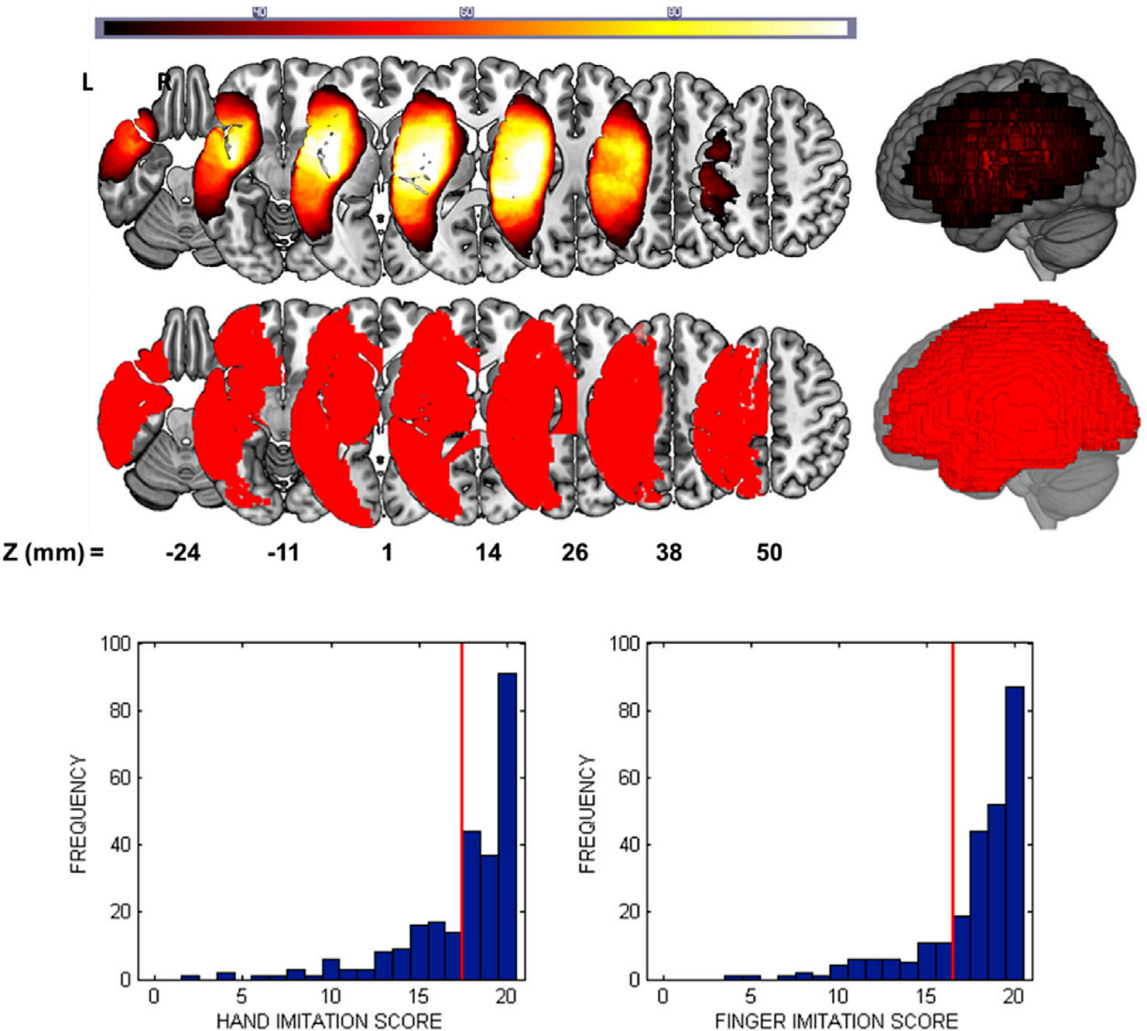
(Top) A lesion frequency image for the 257 patients in our sample. (Middle) An illustration of the union of regions that we consider in the region-based analyses. Both the voxel-based and region-based maps are thresholded to include only those voxels/regions where at least 26 patients (10% of the sample) had damage. (Bottom) Histograms of the patients' scores in the (left) hand and (right) finger imitation tasks. Red lines in each histogram indicate the threshold below which patients were considered ‘impaired’ on that task.

Fig. 1 displays a lesion frequency map for the whole sample of the 257 LH stroke patients, together with histograms of their scores in the hand and finger imitation assessments. There were 85 and 55 patients assessed as impaired on the hand and finger imitation tasks respectively. With respect to behavioural dissociations 40 patients were impaired in the hand task and not the finger task, and 10 patients were impaired in the finger task and not the hand task, though the finger impairment was mild (i.e. scores no more than 2 points below the impairment threshold) in 8/10 of those patients.

### 3.2 Voxel-level analyses

#### 3.2.1 Voxel-based lesion-symptom mapping: Frequentist statistics

**Fig. 2:**
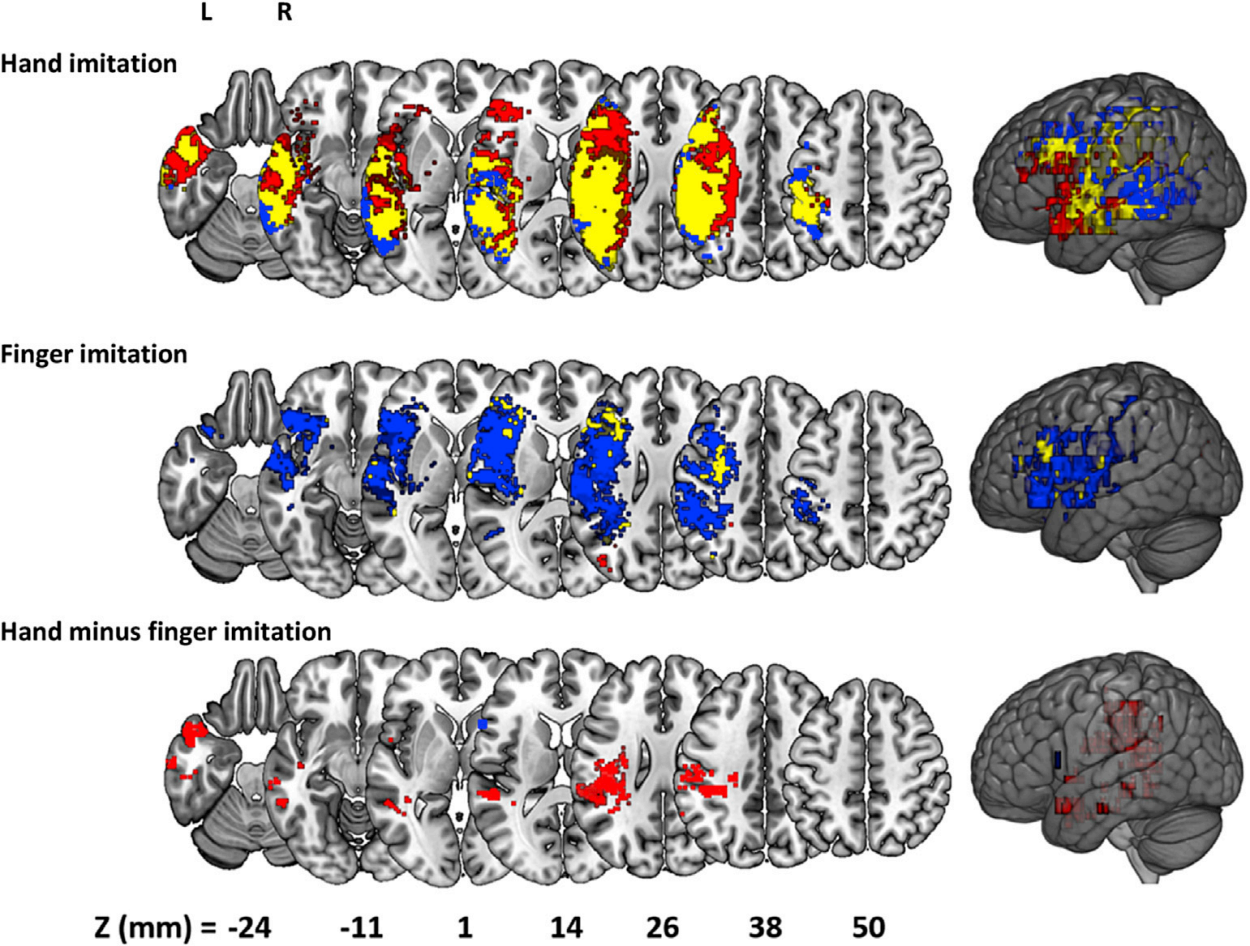
Voxel-based Lesion Symptom Mapping (VLSM) of hand (top) and finger (middle) imitation skills, and of the difference between them (bottom). Red voxels are those that surpassed FDR correction (1%) only prior to controlling task scores for nuisance covariates. Blue voxels surpassed the same threshold only after controlling for nuisance variables. Yellow voxels surpassed the same threshold in both analyses.

Fig. 2 displays the results of the classical VLSM (Bates et al., 2003), both for hand and finger imitation scores, and for the difference between them: all analyses are thresholded at the more conservative threshold of FDR = 0.01 used in (Hoeren et al., 2014). We distinguished between voxels found: (a) only before controlling for the three nuisance covariates (lesion volume, age at stroke onset and time post stroke) (red); (b) only after controlling for the nuisance covariates (blue); and in both cases, i.e. before and after the control (yellow). Consistent with past work, we found fewer significant voxels for finger than hand imitation deficits, and possibly because of the current sample size (n = 257) some voxels survived in every analysis at the same threshold.

**Fig. 3:**
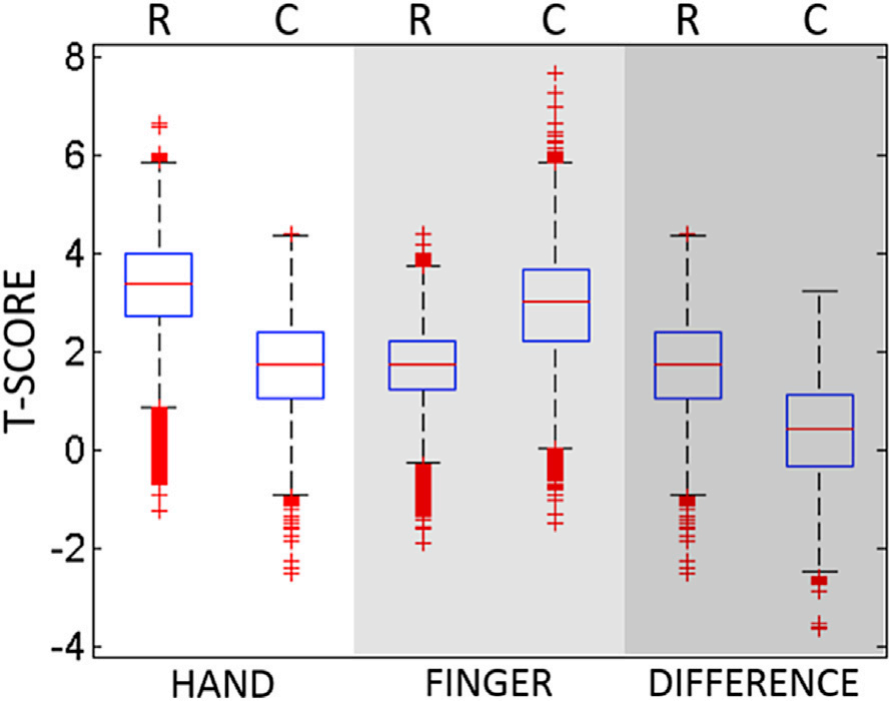
Boxplot of the T-statistics for the VLSM maps in analyses of hand and finger imitation deficits, and of the difference between them. Each analysis is associated with two boxplots, relating to maps generated from Raw (R), and Controlled (C) scores.

Perhaps surprisingly, our control for nuisance covariates had different effects on the individual analyses: reducing the apparent strength of the measured associations in the hand and difference analyses, but increasing that strength in the finger analysis (see Fig. 3). The control appears to have qualitative and quantitative effects: reducing (or increasing) the size of the set of voxels that survive the correction and shifting that set in space. For hand imitation deficits, for example, more anterior voxels survive FDR correction (p < 0.01) before the control is applied, and more posterior voxels survive after the control is applied (Fig. 2top). For finger imitation deficits, the pattern is reversed, with a small posterior cluster surviving before, and a larger anterior set surviving after the control was employed (Fig. 2middle). Finally, in the difference analysis, there was no overlap between the (1,978) voxels which survived the correction before, and the (11) voxels which survived the correction after, the control was employed (Fig. 2bottom).

Although the control for nuisance variables affected the results of our VLSM, there was consistency with regards to both the controlled and uncontrolled analyses which emphasised a role for the inferior parietal lobule (IPL) in hand imitation skills. The sub-region PFt of the IPL (Caspers et al., 2008) contained the highest mean BF in both the controlled and the uncontrolled variants of these analyses, and several other sub-regions were also highly ranked.

At the more conservative FWE correction (p < 0.05), the control's apparent effect is still more extreme. In the finger analysis, voxels only survive the correction after the control is employed, whereas in the difference analysis, voxels only survive the correction before the control is employed. If best practice is to employ controls like ours (e.g. (Karnath and Smith, 2014, Rorden et al., 2007)), then these results throw doubt on past analyses that purport to distinguish hand and finger imitation skills, at the voxel-level, in the LH of the brain (Goldenberg and Karnath, 2006, Hoeren et al., 2014).

#### 3.2.2 Voxel-based lesion-symptom mapping: Bayesian statistics

**Fig. 4:**
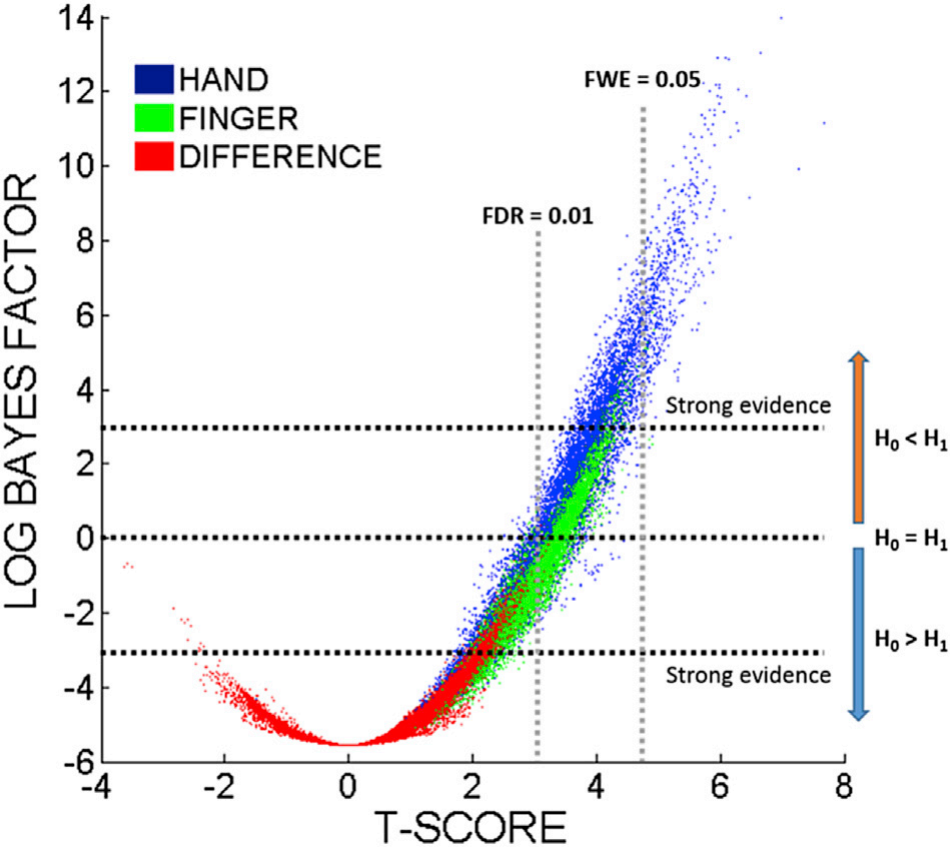
Scatter graph plotting t-statistics against log Bayes Factors in VLSM analyses of hand and finger imitation scores, and of the difference between them for each of the tested voxels. For comparison, we plot the threshold after correction for multiple comparisons using both FDR = 1% (used in the VLSM analyses), and the more stringent FWE = 5% (calculated using permutation thresholding).

To try to clarify the results of the previous section, we repeated the controlled analyses after replacing the *t*-test with a Bayesian ANOVA (as described in the Methods). The results are displayed in Fig. 4, which plots the Bayes Factors assigned to each voxel, in all three analyses (i.e. of hand and finger imitation scores, and of the differences between them), against the t-scores assigned previously. As expected, there is a strong correspondence between the Frequentist and Bayesian VLSM methods, and the qualitative differences between the analyses are preserved: i.e. lesion-symptom associations are stronger for hand than finger imitation, and stronger for both individually than for the difference (i.e. hand minus finger imitation scores) between them. Our threshold for ‘strong evidence’ in favour of an association (BF > 20; log BF >∼3), is more conservative than the FDR threshold used previously (p < 0.01), but more permissive than the FWE threshold (p < 0.05, see Fig. 4).

Consistent with the logic of FDR correction, our Bayesian analysis also assigns negative log Bayes Factors to some voxels which survive the correction at FDR = 0.01. In these cases, our Bayesian test suggests that the evidence against a lesion-symptom association is somewhat stronger than that in favour: these voxels may be false positives. Critically, after applying the control, our Bayesian test also assigns negative log BF to all but 6 of the tested voxels (>99.9%) in the difference analysis, and a maximum BF of just 1.4. In other words, there is no substantial evidence for a difference between the two tasks anywhere in the portion of the brain that we could test, and at least some evidence for *consistency* across the two tasks, almost everywhere in that same set of voxels.

### 3.3 Region-level analyses

Our VLSM results (a) strongly encourage the application of a control for lesion volume (among other nuisance covariates) and when the control is employed, (b) suggest that lesion-symptom associations for hand and finger imitation skills, at least in the LH of the brain, are substantially similar. One interpretation of the results is that we find no differences because there are none, in other words that contrary to past results (Goldenberg and Karnath, 2006), hand and finger imitation skills actually do share the same neural resources, at least in the LH of the brain. On this analysis, variance that is not shared between the two tasks might be explained as an artefact either of noise, or of differential task difficulty, with the latter lent credence because task differences are observed before lesion volume is controlled, and patients with larger lesions tend to have more severe cognitive deficits after stroke, which might particularly impact more difficult skills.

Another interpretation, which we explore in what follows, is that there really are differences between the lesion-symptom associations for these two skills, but that VLSM is not the right way to find them. VLSM is still the most popular way to characterise lesion-symptom associations, but there is increasing recognition that mass univariate methods can fail to capture key associations (e.g. (Inoue et al., 2014, Mah et al., 2014)), largely regardless of the particular statistics employed at each voxel. Here, we test this intuition first by aggregating voxels into regions, as described in the Methods, and then measuring the associations between the proportion of those regions that a lesion destroys, and each of our three target variables (i.e. hand and finger imitation scores and the difference between them, after controlling for nuisance covariates). For ease of comparison with the results of the last section, we use a Bayesian formalism (Wetzels and Wagenmakers, 2012) to quantify the evidence in favour of, and against, the presence of correlations in each case.

#### 3.3.1 Single region analysis

**Fig. 5:**
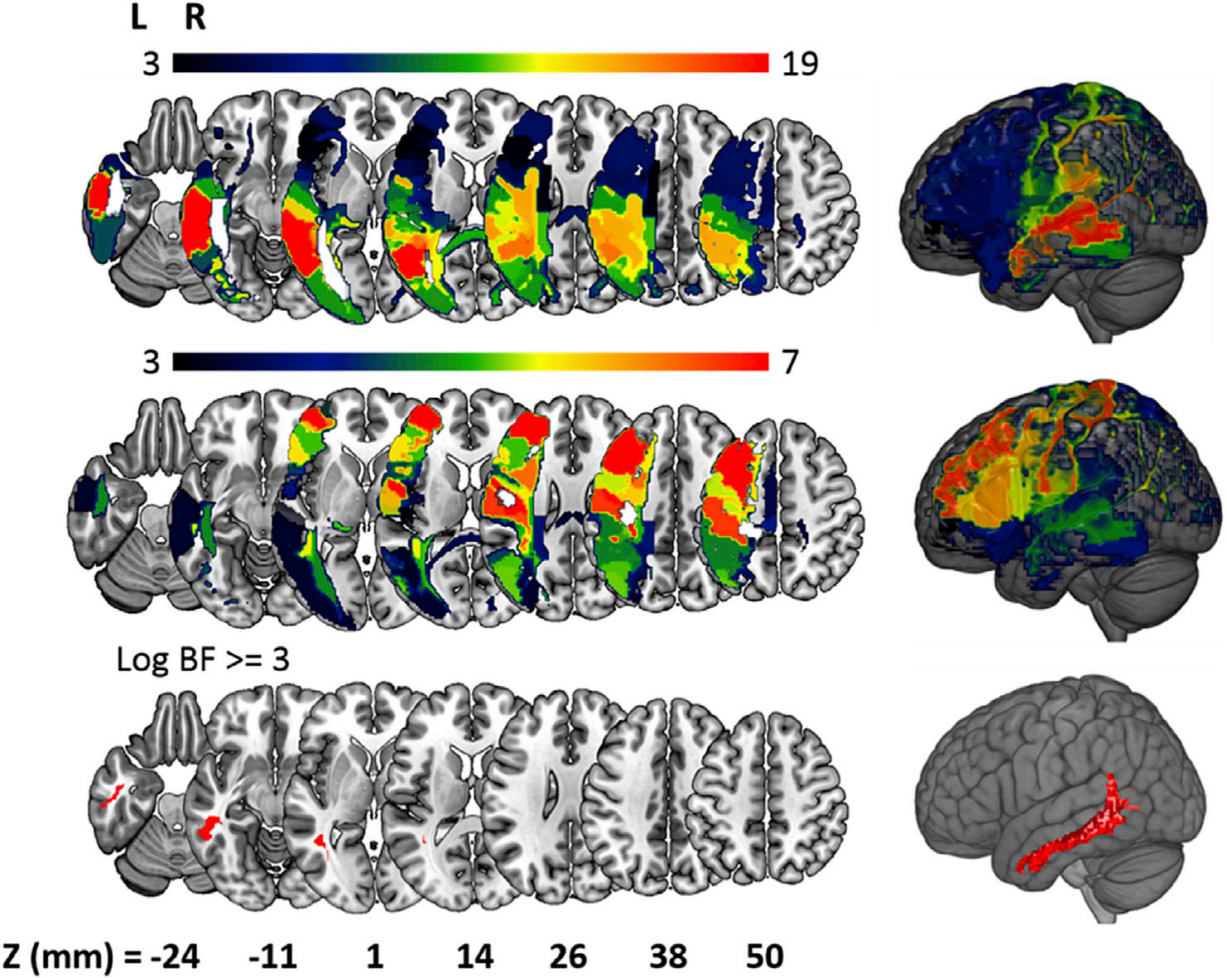
Single-region analyses of hand (top row) and finger (middle row) imitation skills, and of the differences between them (bottom row). Colours in each region reflect the log Bayes Factor assigned to the correlation between lesion load in that region, and the relevant target variable. Regions are only shown when there is a ‘strong’ (BF > 20; log BF > ∼3) association between lesion load in that region and the target variable. The single regions where these associations are strongest are highlighted in white in the hand and finger analyses (top two rows), but one region reaches the threshold in the difference analysis (bottom row).

The single regions results are qualitatively similar to the VLSM results, with strong correlations between the BF assigned to each region, and the peak BF assigned to any voxel in that region, in all three analyses (hand: r = 0.81, p < 0.001; finger: r = 0.75, p < 0.001; difference: r = 0.65, p < 0.001). As with in VLSM, the evidence for lesion-symptom associations was stronger for hand than for finger imitation. This was true both in terms of the number of regions which displayed ‘strong or better’ evidence for associations (hand: 87/122 (71%); finger: 52/122 (43%)), and in terms of the strength of the evidence in those regions (i.e. BFs were higher in the hand analysis than in the finger analysis: see Fig. 5). The most strongly emphasised single region for finger imitation was the primary somatosensory cortex area 3a and the inferior longitudinal fasciculus for hand imitation. With regards to the difference, this analysis yielded weaker associations – again, as we found with VLSM – but there was strong evidence for an association between task score differences and lesion load in the white matter connecting the inferior and medial temporal gyri (BF = 27.2); see Fig. 5. Lesion load in this region was strongly associated with symptom severity for hand imitation deficits (hand: BF > 10^6^), but only ‘substantially’ (Jeffreys, 1961) (BF = 4.6: i.e. marginally) associated with deficits of finger imitation. However, as in the VLSM (and in (Hoeren et al., 2014)), there were no regions where the association between lesion load and finger imitation deficits was significantly stronger than that between lesion load and hand imitation deficits. Finally, every region which appeared to be at all relevant to the differences between task scores (BF > 1) was also relevant to both task scores separately (BF > 3). In other words, there were no regions where damage appeared to affect hand imitation skills and not finger imitation skills, or vice versa.

#### 3.3.2 Region-pair analysis

One of the main motivations for moving from voxels to regions was the recognition that voxel-based mass univariate analyses ignore potentially relevant information. Though our single-region analyses are multivariate in the sense that they aggregate many voxels together, they are also still mass univariate in the sense that we analyse each region in isolation. None of the 122 regions considered here can completely explain the scores in our two imitation tasks (and by implication the differences between them). To take that step, we need to measure how the influence of damage in different regions interacts.

Our approach here was to analyse all of the pairs of the original 122 regions, and to ask whether we can improve each pair by adding an extra (third) region. To make that assessment for a given pair, we calculated the partial correlation coefficients (given lesion load in that pair) for every *other* region, relative to scores in the hand and finger imitation tasks and the differences between them. The intuition here was that ‘better’ region pairs should explain most of the variance in our two scores (and the differences between them), which in turn predicts that these partial correlations will be weak. The better the pair, the weaker will be the strongest partial correlation that we can find. As in the last analysis, we quantified the strengths of these correlations using the Bayesian formalism, due to (Wetzels and Wagenmakers, 2012), converting the maximum partial correlation coefficient into a ‘maximum residual Bayes Factor’ for each region-pair.

**Fig. 6:**
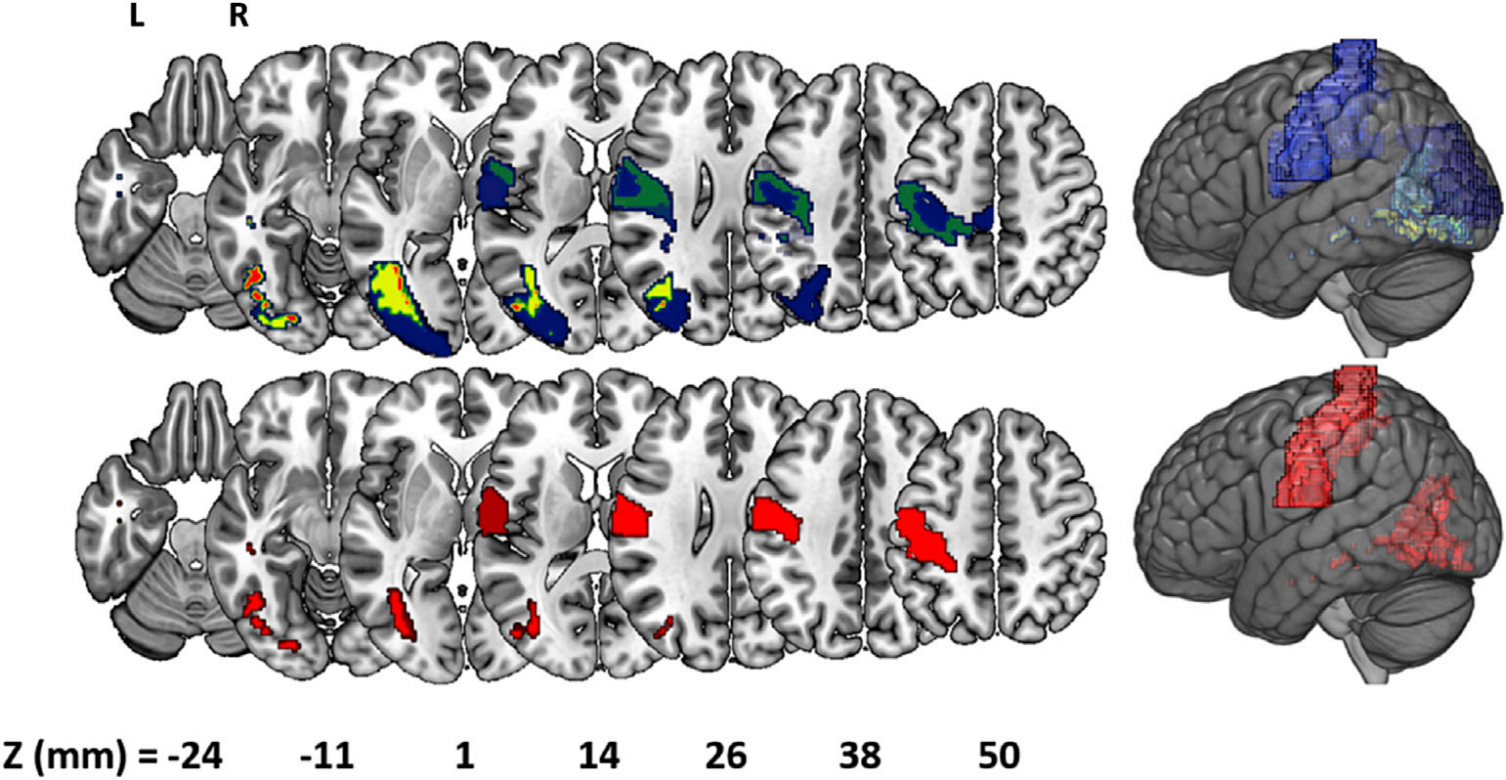
(Top) A frequency image of the regions which appeared in the 9 region-pairs assessed as supplying a ‘sufficient’ account of hand and finger imitation deficits, and of the individual differences between them. The most frequent region – white matter projections between the middle occipital gyrus and the fusiform gyrus (shown in red) – occurred in 5/9 region pairs. (Bottom) The ‘best’ pair of regions identified in the region-pair analysis: this is best explanation of where damage causes both hand and finger imitation deficits in these patients.

Of the 7381 pairs considered (122!/(2! * (122-2)!)), there were 9 cases in which the maximum residual Bayes Factor was <1: i.e. once lesion load in those 9 region pairs was taken into account, no third region could usefully explain any more of the variance in the task scores or the differences between them. These region-pairs each provide a ‘reasonably sufficient’ account of the lesion-symptom associations that drive our behavioural data. The best pairs included: the postcentral gyrus (twice) (Tzourio-Mazoyer et al., 2002), the middle occipital gyrus (Tzourio-Mazoyer et al., 2002), the primary motor cortex (areas 4a and 4p appear in separate region-pairs) (Geyer et al., 1996), areas 3a (twice) and 3b of the somatosensory cortex (Geyer et al., 1999), the fronto-parietal sub-region of the superior longitudinal fasciculus (twice) (Hua et al., 2008), and finally, white matter fibres extending between the middle occipital gyrus and (a) the inferior occipital gyrus (three times), and (b) the fusiform gyrus (five times) (Hua et al., 2008): see Fig. 6 and supplementary Fig. 1. Every one of those regions is also strongly associated (BF > 20) with scores in both the hand and finger scores when considered in isolation: i.e. as in the single-region analysis, damage in all of these regions was associated with deficits in *both* imitation skills.

Notably, the best region-pair shares no voxels at all with any of the three regions which were most strongly emphasised in the single-region analysis (hand: inferior longitudinal fasciculus; finger: primary somatosensory cortex area 3a; difference: white matter connecting the inferior and medial temporal gyri). Just as there was a disconnection between the VLSM results and those garnered from the single-region analysis, so there is a disconnection here between the latter and our region-pair results.

## 4 Discussion

Lesion-symptom analyses of hand and finger imitation deficits, at least in the left hemisphere of the brain, have yielded inconsistent results. Quantitative analyses of large samples of stroke patients have struggled to reproduce the often more qualitative dissociations observed in earlier, smaller-scale studies. Armed with perhaps the largest single sample yet studied in this area, we have attempted both to explain that inconsistency, and to resolve it.

We have employed two methodological innovations in this work: a Bayesian VLSM, adapted from the Bayesian ANOVA proposed in (Wetzels et al., 2012), and a Bayesian correlation and partial correlation analysis, as developed in (Wetzels and Wagenmakers, 2012). Both are useful because they allow us to quantify the evidence for *and* against the tested association. In the VLSM, the Bayesian formalism helped us to understand why no significant voxels were found in the difference analysis, and in the region-pair analysis it served to naturally distinguish those pairs that would benefit from the addition of further regions (i.e. whose maximum residual Bayes Factor was >1), from those that would not benefit. In the case of the VLSM, there is an extra benefit because Bayes Factors are arguably meaningful irrespective of corrections for multiple comparisons (Friston et al., 2002).

Our VLSM results are largely consistent with prior research. First, both before and after controlling for the nuisance covariates, our VLSM results emphasised a role for the inferior parietal lobule (IPL) in hand imitation skills. The sub-region PFt of the IPL (Caspers et al., 2008) contained the highest mean BF in both the controlled and the uncontrolled variants of these analyses, and several other sub-regions also appeared near the top of both ranked lists. The IPL has long been reported to be involved in imitation and apraxia (Liepmann, 1920), and is still widely acknowledged to play an important role (Brass and Heyes, 2005), with evidence both from studies of apraxic patients with LH stroke (Alexander et al., 1992, Basso et al., 1987, Dovern et al., 2012, Haaland et al., 2000, Halsband et al., 2001, Kertesz and Ferro, 1984), and from a recent Activation Likelihood Estimation (ALE) meta-analysis of 139 fMRI and PET studies of neurologically normal participants (Caspers et al., 2010) as well as studies adopting neuromodulation (Weiss et al., 2013). In the finger imitation analysis, before controlling for nuisance covariates, the VLSM yielded peak voxels in the left posterior corona radiata, and in the white matter beneath the superior parietal lobule, as well as in the inferior parietal lobule. This is consistent with the regions implicated (albeit at a permissive FDR threshold) in the equivalent analysis in (Hoeren et al., 2014) and similar to the results reported in Dovern et al. (2011). Finally, we found no evidence for the anterior (finger)/posterior (hand) dissociation reported in some earlier studies of these tasks (Haaland et al., 2000), consistent with the larger and more recent study by Hoeren and colleagues (Hoeren et al., 2014).

Our analysis suggests that many of these voxel-level results are confounded by lesion volume. A control for it did not simply decrease the lesion-symptom ‘signal’ across the brain: the signal was reduced in the hand imitation analysis, but increased in the finger imitation task. In both cases, some voxels survived FDR correction only before, and others survived only after the control was applied. Critically, the application of the control largely extinguished any apparent differences between the two tasks at the level of single voxels – as in (Hoeren et al., 2014), where the same control had similar effects. Armed with a Bayesian formalism, we can interpret this result as real evidence *against* a task dissociation, rather than just as a lack of evidence in favour.

The single regions results were qualitatively similar to the VLSM results. Those regions where the peak voxels occurred in each VLSM analysis displayed similar damage-deficit associations when considered as a whole: *hand*: area 2 of the primary somatosensory cortex (Grefkes et al., 2001), single region level BF > 10^4^; *finger*: OP4 of the parietal operculum (Eickhoff et al., 2007), single region level BF = 52; *difference*: posterior superior longitudinal fasciculus, single region level BF = 4. However, in each analysis, the strongest region-level associations were found elsewhere: the region-level and voxel-level analyses emphasised different parts of the brain. And in the case of the ‘difference analysis’, there was another and arguably more important difference. In the VLSM, no voxel displayed evidence strong enough to be ‘worth more than a bare mention’ (BF > 3: (Jeffreys, 1961)) in favour of a task dissociation (maximum BF = 1.4), but the region-level analysis yields strong support (BF = 27) for that dissociation, associated with lesion load in the white matter projections between the inferior and middle temporal gyri (Hua et al., 2008). Almost all of the voxels contained within that region mask were analysed individually as part of the voxel-based analyses. This is evidence that the aggregation itself (i.e. of voxels into regions) is driving the differences between these levels of analysis.

The single region analyses might be considered preferable to the VLSM analyses, because they take account of interactions between voxels that will otherwise be ignored. But the single-region analyses also arguably offer too much of a good thing by implicating far more regions than they should: i.e. they appear to display poor specificity. This is important when we are trying to find regions where damage affects one skill but *not* the other – because inflated lesion-deficit associations will mask regions where damage is not associated with deficit. Our region-pair analyses offer a potential solution to this problem, by focusing on the unique contribution a region makes, once damage in other regions is taken into account.

As in most neuroscience studies, theory in our study is under-determined by the data; no single region-pair provides so much better an account of the data than any other that we can confidently select that pair above all others. But the regions implicated in the ‘reasonably good pairs’ are all quite consistent (see Fig. 6, top row): they encourage an emphasis on the occipital lobe, extending into the temporal lobe (potentially consistent with the task dissociation reported in the lateral occipito-temporal cortex in (Hoeren et al., 2014)), and on the primary motor and somatosensory cortices. Moreover, though the results of the region-pair analysis are somewhat equivocal, because we cannot select a single region-pair with real confidence, they do imply an unequivocal answer to one of the questions that first motivated this work: if and how hand and finger imitation skills are dissociated in the left hemisphere of the brain. Our best accounts of hand and finger imitation deficits all refer to regions where damage seems to affect both skills.

Damage in those region-pairs does tend to affect hand imitation skills more than finger imitation skills; this relationship is explicit in the regression coefficients (b-values) of our best region-pair models, which are consistently larger (more negative) for hand than finger imitation scores. For example, in our best model, with regions illustrated in Fig. 6, the regression coefficients are: hand = 0.28 + (Reg1 x −1.17) + (Reg2 x −1.05); finger = 0.28 + (Reg1 x −0.91) + (Reg2 x −0.76). All of the other 8 ‘reasonably good’ region-pair models (Fig. S1) follow the same pattern. Though these differences do not rise to the level of a significant task × lesion interaction in any of the region pairs (analyses of variance; all p > 0.1), they are nevertheless consistent with the pattern of results reported by Hoeren and colleagues (Hoeren et al., 2014), who found voxels where lesion damage affected hand significantly more than finger imitation skills but not vice versa, and may help to explain why 40 of our patients were assessed as having hand imitation deficits with finger imitation skills in the normal range.

That being said, our group-level results do not purport to account for every individual patient's deficit profile: indeed, 5 patients assessed as suffering from an apparently selective gesture imitation deficit (i.e. hand or finger but not both) had no damage at all in either of the brain regions implicated by our ‘best’ region pair (Fig. 6). We do not dismiss these patients' deficits as being somehow artefactual: we simply find no simple, consistent way to account for them in terms of the lesion damage that they have suffered. This might be because these patients' lesions are simply unusual, or because the effects of those lesions are inconsistent – perhaps the result of unusual, premorbid organisation of these skills in the brain. In that case, even group analyses with huge sample sizes will fail to identify those lesion-symptom effects. This logic might also apply to the 10 patients who appeared to have finger but not hand imitation deficits: i.e. the reverse pattern of effects that our neuroanatomical results might predict. 8/10 of these patients had only very mild finger imitation deficits, which might in principle be modelled as noise in the model. But this logic cannot easily account for the remaining two patients, whose finger imitation deficits were moderate or severe with apparently preserved hand imitation skills.

Finally, as mentioned previously, the finger imitation task likely engages a more complex network of (semantic and non-semantic) processes (Achilles et al., 2016) than the hand imitation task. This might explain some of the wider variability between the hand and finger imitation scores. But whatever the truth, our group-level analyses suggest that effector-specific lesions are either rare or inconsistent in their effects, or both. The simplest, most powerful lesion-symptom models that we found for these data make no significant distinction between hand and finger imitation skills in the brain.

None of the regions ranked most highly in any of the three single-region analyses appear in any of the best region pairs that we found: there is a disconnect here, analogous to that between the single-region analyses and the VLSM. This disconnect is important in the context of recent studies which aim to characterise the functional role of individual (often anatomically defined) brain regions in much the same way as we did in the single-region analysis – either singly (e.g. (Tak and Jang, 2014)) or in small groups of two or three (e.g. (Hope et al., 2015b, Marchina et al., 2011)). Our results suggest that individual brain regions can be given ‘credit’, in these analyses, which they do not necessarily deserve. Every single region that we considered had a positive maximum residual Bayes Factor. This means that every single-region model of the data could sensibly be improved by considering lesion load in at least one other region – and our best two-region models were better than any single-region model that we found. But in making the move from one region to two, we found that regions which seemed critical when viewed in isolation did not seem so important after all once the rest of the brain (or the hemisphere) was taken into account.

The regions actually implicated by our results – the primary somatosensory and motor cortices, typically paired with regions in and around the occipital cortex – are consistent with accounts of imitation which emphasise direct sensor-motor connections (Brass and Heyes, 2005, Gonzalez Rothi et al., 1991). Our best pairs include regions from two different atlases which emphasise the primary somatosensory cortex (on the postcentral gyrus), and also implicate the primary motor cortex. Though the motor system appears to be crossed, with left hemisphere regions driving contralateral (right-sided) movement and vice versa, motor damage in the left hemisphere has been observed to impair movement with the ipsilateral (left) hand (Wyke, 1971), and neurones in left premotor and motor regions have been found which appear to be tuned to movement with the left hand (Cisek et al., 2003). Most of the other regions which appear in those ‘best’ region-pairs are in the occipital lobe, associated with visual perception in general and also specifically with the perception of hand and finger gestures (Hermsdorfer et al., 2001).

Our results are harder to reconcile with accounts of imitation which imply a special role for a ‘common code’, interposed between the perception and reproduction of hand and finger gestures. In particular, when the combinatorics of brain damage in pairs of regions are considered we find no special role for the IPL, which is implicated as the locus of the critical processing under the body-part-coding hypothesis (Goldenberg and Karnath, 2006). That region (among others) is implicated in the single-region analysis of the task differences (Fig. 5, bottom row), and also in the VLSM analyses presented here – and many prior studies, including some of our own, support some critical role for this region in gesture imitation (Brass and Heyes, 2005, Alexander et al., 1992, Basso et al., 1987, Dovern et al., 2012, Haaland et al., 2000, Halsband et al., 2001, Kertesz and Ferro, 1984, Caspers et al., 2010, Weiss et al., 2013). Nevertheless, the region-pair results suggest that this region might not be as critical as previously thought. One implication of this result might be that damage to the IPL, or indeed to any other single region which does not appear in more complex models, is only contingently correlated with the damage that really causes these deficits.

One caveat to these results flows from the wide range of times post-stroke at which our patients were assessed: some of our patients may have recovered from initial gesture imitation deficits, while others have not had time to recover (58% were assessed < 1 month post-stroke). This might inject additional noise into our data, making otherwise consistent lesion-symptom associations more difficult to find. And though we used time post-stroke as a nuisance covariate to mitigate this effect, that noise might still be masking important lesion-symptom associations.

Another caveat is that, though well motivated, the line that we have drawn between the nine ‘reasonably sufficient’ region-pairs and the rest is still rather fine. In our results, that line is drawn where the maximum residual Bayes Factor for a given pair is less than ‘1’ – where the evidence that the pair might be improved by adding a third region is greater against than in favour. But there are a lot (78) of other region-pairs where the evidence that they can be improved is stronger than that against, but still very weak, or ‘not worth more than a bare mention’ (i.e. maximum residual Bayes Factor <3) (Jeffreys, 1961). Some (11) of these region-pairs do include the IPL, and the best of those (which pairs the IPL with the inferior longitudinal fasciculus; rank = 16) has a maximum residual Bayes Factor of just 1.32. In other words, while our results give greater support to purely or mainly sensor-motor accounts of imitation, than to accounts which emphasise intermediate processing (e.g. in the IPL), we find that both accounts can offer at least a potentially reasonable account of the data.

Finally, we do not claim that region-pairs are the best or only sensible way to characterise the critical lesion-deficit associations for gesture imitation skills. In recent years, we and others have proposed much more overtly multivariate methods than those considered here (Hope et al., 2013, Hope et al., 2015a, Kuceyeski et al., 2015, Rondina et al., 2016, Yourganov et al., 2016), to characterise these associations. One recent study in this vein showed that multivariate models driven by collections of voxels were better able to predict motor impairments after stroke than models driven by collections of (anatomically defined) regional lesion load variables (Rondina et al., 2016). This trend may extend to gesture imitation deficits too. We chose to focus on VLSM here to maximize the correspondence with prior work, and we used region-based analyses principally to explain and expand on those voxel-based results. But this is a rapidly evolving field; there is still no consensus on the best way to capture these associations in general. And one of the main lessons that our results can teach, is that the substantive results of analyses can and do change as we ascend levels of complexity in the models we test: our voxel-based, single-region and region-pair analyses all emphasised different brain regions here. There is every reason to allow that the conclusions we reach may continue to change as our methodology changes.

In summary, we have demonstrated that: (a) in LH stroke patients, there is good evidence *against* a dissociation between hand and finger imitation skills at the voxel-level (when control for nuisance covariates is applied); (b) different levels of analysis for lesion-symptom associations (voxels, regions, and region-pairs) can yield inconsistent results; but also that (c) all three levels suggest that hand and finger imitation skills share similar neural resources in the left hemisphere, with the former being more sensitive to damage which affects both skills. Finally (d), when the combinatorics of damage in pairs of regions is taken into account, these deficits appear to be best explained as driven by damage in the occipital lobe and primary somatosensory/motor cortices. This last result argues in favour of an account of imitation which emphasises direct sensor-motor connections, apparently without the need to posit a ‘common code’, interposed between the perception and reproduction of bodily gestures.

## Funding

This work was funded by Köln Fortune (242/2014) and the Wellcome Trust (097720).
