# Supplementary material for: Using multi-level Bayesian lesion-symptom mapping to probe the body-part-specificity of gesture imitation skills
Source: Neuroimage. 2017 Nov 1;161:94–103. doi: 10.1016/j.neuroimage.2017.08.036 (PMC5692920; doi:10.1016/j.neuroimage.2017.08.036)

**Supplementary Material**

**Figure S1:** Axial slices and rendered views of the region-pairs which each provide ‘reasonably sufficient accounts’ of 257 stroke patients’ gesture imitation deficits.


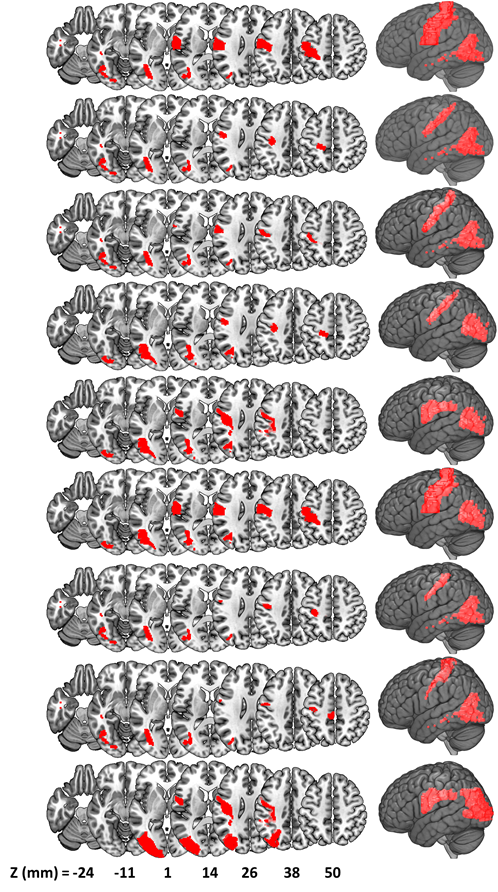

Supplement: Supplementary file 1 [file mmc1.docx]
